# Trends in the Utilization of Ankle Replacements: Data From Worldwide National Joint Registries

**DOI:** 10.1177/10711007211012947

**Published:** 2021-06-17

**Authors:** Thomas A. Perry, Alan Silman, David Culliford, Lucy Gates, Nigel Arden, Catherine Bowen, and the International Ankle Arthroplasty Registry Consortium

**Affiliations:** 1 Nuffield Department of Orthopaedics, Rheumatology and Musculoskeletal Sciences, Botnar Research Centre, University of Oxford, Old Road, Oxford, United Kingdom; 2 Centre for Sport, Exercise and Osteoarthritis Versus Arthritis, Nuffield Department of Orthopaedics, Rheumatology and Musculoskeletal Sciences, University of Oxford, Oxford, United Kingdom; 3 NIHR Applied Research Collaboration (ARC), Wessex, School of Health Sciences, University of Southampton, Southampton, United Kingdom; 4 School of Health Sciences, Faculty of Environmental and Life Sciences, University of Southampton, Southampton, United Kingdom; 5 Centre for Sport, Exercise and Osteoarthritis Versus Arthritis, University of Southampton, Southampton, United Kingdom; 6 MRC Lifecourse Epidemiology Unit, Southampton General Hospital, University of Southampton, Southampton, United Kingdom

**Keywords:** ankle replacement, registries, joint arthroplasty, registry-level

## Abstract

**Background:**

Over the past decade, there has been a growth in the use of ankle replacements. Data from national joint registries have shown between-country differences in the utilization of ankle replacement. The reasons for these differences are, however, not well understood. Our aims were to describe and compare the annual incidence of primary ankle replacement between countries and, to examine potential reasons for variation over time.

**Methods:**

We used aggregate data and summary statistics on ankle replacements for the period 1993 to 2019 from national joint replacement registries in Australia, Finland, New Zealand, Norway, Sweden and the United Kingdom. From the annual recorded counts of procedures, demographic data were extracted on age, sex distribution, and indication(s) for primary ankle replacement. Registry-level summary results were also obtained on data completeness, counts of hospitals/units, and health care providers performing ankle replacements annually and data collection processes (mandatory vs voluntary). Annual ankle replacement incidence for all diagnoses and, by indication categories (osteoarthritis [OA] and rheumatoid arthritis [RA]), were calculated per 100 000 residential population aged ≥18 years.

**Results:**

For the period with data from all 6 countries (2010-2015), New Zealand had the largest annual incidence (mean ± SD) of 3.3 ± 0.2 ankle replacement procedures per 100 000 population whereas Finland had the lowest incidence (0.92 replacements). There were no common temporal trends in the utilization of ankle replacements. Over the years studied, OA was the predominant diagnosis in the United Kingdom, Australia, and New Zealand, whereas RA was the most common indication in Scandinavia.

**Conclusion:**

In these 6 countries, we found marked differences in the utilization of ankle replacements. Registry-related factors including data completeness and the number of hospitals/surgeons performing ankle replacements are likely to contribute to the observed between-country differences and need to be carefully considered when interpreting comparisons for this less common site for joint replacement surgery.

**Level of Evidence:**

Level III, retrospective study.

## Introduction

Osteoarthritis (OA) is a global health burden and is a leading cause of pain, loss of function and disability. Ankle OA often develops following ankle trauma^4,20,56^ with post-traumatic ankle OA occurring in up to 70% of injuries.^49^ Ankle arthrodesis, or joint fusion, is an effective surgical treatment for reducing pain and improving function. There are, however, data that suggest that fusion of the joint following ankle trauma results in the loss of mobility and increases stress on adjacent joints at the foot that can consequently lead to pain, disability, and OA at adjacent joints.^5,17^ Subsequently, new treatment modalities have been introduced for the surgical management of ankle trauma and end-stage ankle OA.

Total ankle arthroplasty, more commonly called total ankle replacement (TAR), was first introduced in the early 1970s.^62^ As early evidence showed high rates of postoperative complications, readmission,^63^ and revisions,^8,9,13,30,37,47,48^ ankle arthrodesis remained the more common surgical treatment for end-stage ankle OA.^42^ Indications for TAR include primary and post-traumatic OA, but also end-stage disease secondary to inflammatory disorders such as rheumatoid arthritis (RA).^24^ The introduction of newer replacements throughout the 1980-1990s permitted more natural movement of the ankle joint,^62^ and there is some evidence to suggest that TAR may show improved postoperative outcomes compared to ankle arthrodesis.^39,47,48,61^ Despite evidence supporting improvement in postoperative outcomes, it remains unclear as to how much TAR has been adopted globally for the treatment of ankle trauma and primary/secondary arthritis.

With most data, but not all,^26^ supporting the effectiveness of TAR, there is growing interest in whether the volume (total number of TAR performed annually) and/or national per capita utilization (standardized to a countries population) of TAR has changed over time and between countries because of demand. More importantly, there are few data examining the primary indications for ankle replacement and changes in these over time; there are only limited epidemiologic studies, which do not use a standardized approach. Thus, alternative data sources have been used to compare incidence rates of this procedure. The usefulness of national joint replacement registry data as a source of information for comparing arthroplasty rates at joint sites and examining replacement safety has been well documented^29,43,45^; however, these data have been underutilized for examining temporal trends in the use of ankle replacements. In recent years, several countries have adopted national joint replacement registries, which allow the long-term surveillance of replacements and allow the assessment of surgical performance. Six countries have previously reported the results of ankle replacements for national joint registries.^11,18,19,21,25,51,63^

A more in-depth and comparative analysis of the uptake of ankle replacements and assessing how disease indications are changing over time would help to inform the national debate on the provision of orthopedic and rheumatologic care. We aimed to report and compare annual incidence rates of primary ankle replacements between national joint registries and examine sources of variation.

## Materials and Methods

We used the findings of a recent systematic review^6^ and the membership list of the International Society of Arthroplasty Registries^22^ to identify all arthroplasty registries collecting national data on primary ankle replacements.

Routinely collected data on ankle replacements were identified and included from the following countries and/or national registries: (1) National Joint Registry for England, Wales, Northern Ireland, the Isle of Man and the States of Guernsey (UK-NJR)^34,35^; (2) Australian Orthopaedic Association National Joint Replacement Registry (AOANJRR)^2^; (3) New-Zealand Orthopaedic Association Joint Registry (NZOA Joint Registry)^36^; (4) Finnish Arthroplasty Register (FAR)^12^; (5) The Swedish Ankle Registry (SwedAnkle)^57^; and (6) The Norwegian Arthroplasty Register.^59^ For this analysis, only data on primary ankle replacements were included.

We obtained the most current aggregate data and summary statistics (ie, published, electronically available yearly annual reports or summary tables) either from the registry websites (online data correct as of January 1, 2021) and/or by direct contact with principal investigators, information technology (IT) teams, and/or orthopedic centers of the corresponding registries for up to December 2019 or registry termination. Data that were not open access was requested from the host registry. The published data from the registries varied in their use of descriptive statistics, with some using medians (IQR) and others means (SD). To allow harmonization, we therefore requested the relevant data (such as age at time of surgery) to be supplied with the same descriptors (eg, mean ± SD).

We sought to examine a range of demographic factors including age, sex, body mass index (BMI), and physical status at time of surgery; however, standardized collection of these data were not available in all registries. Subsequently, we identified the following common variables and extracted only preoperative patient characteristics that were available from all registries: age at surgery, sex distribution, and indication(s) for primary ankle replacement.

Further, data were extracted on registry characteristics including calendar period of data capture (from time of first recording ankle replacement), local registry collection rules (mandatory vs voluntary), and other aspects. We also extracted data on registry completeness; each registry compares the number of ankle replacements recorded to the number recorded in national secondary care databases. In 5 of the 6 registries, annual data completeness ranged from 85% to 100% (median, range); (1) United Kingdom (93%, 0-100), (2) Australia (98%-99%), (3) New Zealand (>95%), (4) Norway (89.8%, 81.1%-96.9%), and (5) Sweden (100%). The Finnish situation was more complex. Whereas data were available from 1980 in Finland, data on annual completeness were only available from 1996 until registry termination in 2015. For Finland, we used data from 1999 onward when data completeness first exceeded 85%.

We examined the temporal trends of the number of hospitals (or units) that performed ankle replacements, including, where available, the annual number of health care providers (eg, surgeons) performing ankle replacements. These data were also extracted from freely available annual reports or were provided by the host registry on request. Additional details regarding the individual joint registries, including methods of data compliance and completeness, are presented in Supplementary Material. Annual counts were based on a calendar year (ie, January 1 to December 31) in all registries.

## Statistical Analysis

We included all ankle replacements (ie, partial: 1 or 2 joint compartments are resurfaced; and total ankle replacements: whole joint replaced) in our total count (Supplementary Table S1). The terms used to capture the indications for primary ankle replacements and the coding of the indications themselves varied between the registries. In an effort to standardize the indications between the 6 registries, we categorized the indications into 4 main groups: (1) OA, (2) RA, (3) trauma, and (4) “other” (Supplementary Table S2). We exclusively examined total procedures (all diagnoses) and, ankle replacements by OA and RA indications. Only Finland, as “primary arthroses,” and Sweden, as “primary OA” and “post-traumatic OA,” differentiated primary and secondary OA.

### Annual Incidence Rate

For each registry, we calculated annual incidences of primary ankle replacements, for all diagnoses and then by OA and RA indications, per 100 000 residential inhabitants using yearly population counts as the denominator.^3,50,52-55^ Where available, we used end of year (January or December) population statistics for residential populations; only mid-year estimates were available for the United Kingdom. The denominator for the UK-based estimates included England, Wales, and Northern Ireland nations only (excluding Scotland) as the UK-NJR does not capture ankle replacements in Scotland. Further, our denominators only comprised residential populations aged ≥18 years. In addition, we also calculated 5-year moving averages (2 lag and 2 lead terms) for the annual incidence rates per 100 000 population for all diagnoses, OA-specific and RA-specific ankle replacements, respectively.

Owing to differences in registry inception dates, we also examined time trends during a period that was common in all registries (2010-2015). Inevitably, in the early years of each joint site-specific register, there was likely to be an exponential growth with increasing uptake of the new technology and completeness of ascertainment. We therefore aimed to identify if there was a peak year in annual incidence and, hence, we also calculated ankle replacement annual incidences in 5-year bands after the year of peak incidence (if a peak was observed).

## Results

### Temporal Trends in Ankle Replacement Utilization

**Figure 1.**
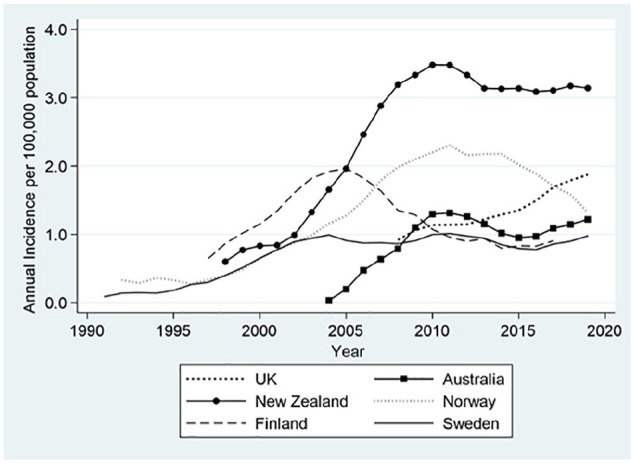
Annual incidence of primary ankle replacement (all diagnoses) for 6 countries. The data represent 5-year moving averages for the annual incidence rates of ankle replacements per 100 000 population for the entire registry period.

In all registries, with the exception of the United Kingdom, there was a greater than linear growth in ankle replacement utilization within the first 5-10 years of registry inception. There was, however, not a similar secular trend of utilization of ankle replacements (Figure 1). There were marked differences in rates of ankle replacements from the year of peak incidence with different time trends observed in the different registers. These trends included a gradual but continuous decline in the number of replacements (Norway and Finland), a gradual decline followed by a plateau (New Zealand), a gradual decline followed by a second growth in replacements (Australia) and no discernible pattern (Sweden). In addition, the UK NJR was the only registry to show a continuous growth in the number of ankle replacements (Figure 1).

### Annual Incidence of Ankle Replacements

Taking into consideration the entire registry period, New Zealand had the greatest annual incidence of ankle replacements per 100 000 population whereas Sweden had the lowest annual incidence (Table 1).

**Table 1.** Annual Incidence Rates for Primary Ankle Replacement—All Diagnoses.

Country	Annual Incidence Rates for Primary Ankle Replacement per 10^5^ population (All Diagnoses)						
	Years Included	Year of Peak Annual Incidence^a^	5-Year Moving Averages for Entire Registry Period	5-Year Moving Averages for Overlapping Period^b^	5-Year Moving Averages After Year of Peak Incidence		
					0-4 y	5-9 y	≥10 y
Australia	2006-2019	2011	0.9 ± 0.4	1.2 ± 0.2	1.1	1.1	–
Finland	1999-2015	2005	1.2 ± 0.4	0.9 ± 0.1	1.6	0.9	0.8
New Zealand	2000-2018	2010	2.4 ± 1.1	3.3 ± 0.2	3.3	3.1	–
Norway	1994-2019	2009	1.3 ± 0.7	2.2 ± 0.1	2.3	1.9	1.3
Sweden	1993-2019	3x peaks: 2003, 2011, 2019	0.7 ± 0.3	0.9 ± 0.1	0.9^c^	1.0^c^	0.9^c^
United Kingdom	2010-2019	No peak	1.3 ± 0.3	1.2 ± 0.1	1.2 ^d^	1.6 ^d^	–

All results are presented as means ± SD.

a Year of peak annual incidence was based on raw annual incidence rates per 10^5^ population for all diagnoses.

b Overlapping period is defined as the period of 2010 to 2015 where all 6 registries had data.

c Sweden had 3 peaks and so we used the first peak (2003) as the year of peak incidence.

d The United Kingdom did not show a year of peak incidence, and so we present 5-year moving averages from registry inception (ie, 2010).

The combined mean annual incidence for the period with data from all registries (2010-2015) was 1.6 procedures per 100 000 population. New Zealand was found to have the largest annual incidence per 100 000 population, and Finland reported the lowest incidence (Table 1). After each registry’s respective peak, with the exception of the United Kingdom, which has yet to reach peak incidence, and Sweden, which had 3 peaks, the other 4 registries showed a gradual decline in 5-year moving average annual incidence rates. Norway showed the steepest decline in incidence with an overall change in annual incidence of −0.9 ankle replacements per 100 000 population.

### Differences in Registry-Related Factors

Registry-level data are summarized in Table 2. After each country’s data allowed for differences in the size of the population, New Zealand was found to have the greatest number of hospitals/units performing ankle surgery per 100 000 population whereas Sweden had the fewest hospitals/units performing ankle replacement. Similarly, New Zealand had the greatest number of surgeons performing ankle surgery per 100 000 population whereas Sweden had the fewest number of surgeons performing ankle surgery. Annual counts of surgeons performing ankle replacements were not reported for Finland and Norway.

**Table 2.** Characteristics of the Included National Joint Registries.^a^

Country	Years Included	Number of Hospitals or Units Performing Ankle Replacement per Year			Mean Number of Hospitals/Units Performing Ankle Replacement per 100 000 Population	Number of Consultants Performing Ankle Replace-ment per Year	Mean Number of Consultants Performing Ankle Replacement per 100 000 Population	Data Capture
		Public	Private	Total				
Australia	2006-2019^b^	17 (9-22)^c^	38 (2-47)	56 (2-67)	0.3	42 (2-53)	0.2	Voluntary
Finland	1999-2015^d^	8 (4-12)	1 (0-2)	8 (5-12)	0.2	–	–	Voluntary
New Zealand	2000-2019^e^	10 (3-15)	10 (3-15)	21 (7-28)	0.6	15 (7-21)	0.4	Mandatory^f^
Norway	1994-2019^g^	6 (2-10)	0 (0-1)	6 (2-11)	0.2	–	–	Voluntary
Sweden	1993-2019^h^	7 (1-11)	1 (0-5)	9 (1-13)	0.1	8 (1-10)	0.1	Voluntary
United Kingdom	2010-2019^i^	–	–	143 (104-155)	0.3	139 (107-156)	0.3	Mandatory^f^

a All results are presented as median values (range: minimum and maximum values) unless otherwise stated.

All values relate to primary ankle replacement unless otherwise stated.

b Data provided directly from the Australian Orthopaedic Association National Joint Replacement Registry.

c Data available for 2007-2019.

d Data provided directly from Finland Arthroplasty Register.

e Data provided directly from New Zealand Orthopaedic Association.

f Compulsory: unless the patient does not provide informed consent for data collection.

g Data provided directly from Norway Arthroplasty Register.

h Data provided directly from SwedAnkle.

i Data available at https://reports.njrcentre.org.uk and https://reports.njrcentre.org.uk/Portals/0/PDFdownloads/NJR%2017th%20Annual%20Report%202020.pdf

### Demographics of Patients Undergoing Ankle Surgery

Demographic data are reported in Table 3. For the period 1993-2019, the total number of primary ankle replacements recorded within the 6 registries exceeded 14 000 (N = 14 675). For primary ankle replacements (all diagnoses), mean age at surgery ranged from 57.3 to 68.5 years, and men and women undergoing surgery were of a similar age. Furthermore, there were marked differences between the sexes: primary ankle replacements were more common in men in the United Kingdom and Australasia but were more frequent in women in Scandinavia.

**Table 3.** Patient Demographics of Primary Ankle Replacements Across National Joint Registries.

Country	Years Included	Total Count, n	Sex, Female, %	Age, y, mean ± SD		
				Men	Women	All
Australia	2006-2019^a^	2564	40.3	67.3 ± 8.7	66.0 ± 9.9	66.8 ± 9.2
Finland	1999-2015^b^	935	62.0	57.8	57.3	57.5
New Zealand	2000-2019^c^	1737	39.4	66.9 ± 8.3	63.5 ± 9.5	65.5 ± 9.0
Norway	1994-2019^d^	1310	53.9	61.8 ± 11.6	60.1 ± 13.2	60.6 ± 12.8
Sweden	1993-2019^e^	1460	59.0	60.4 ± 11.3	58.9 ± 12.3	59.6 ± 12.0
United Kingdom	2010-2019^f^	6669	40.9	68.5 ± 9.3	67.0 ± 11.2	67.8 ± 10.2

a January 2006–December 31, 2019: data directly provided from Australian Orthopaedic Association National Joint Replacement Registry.

b 1999-2015: data provided directly from Finland Arthroplasty Register.

c January 2000–December 2019: data provided directly from New Zealand Orthopaedic Association.

d 1994-2019: data provided directly from Norway Arthroplasty Register.

e 1993–December 31, 2019: data provided directly from SwedAnkle.

f April 1, 2010–December 31, 2019: data available at https://reports.njrcentre.org.uk/

### Temporal Changes in Primary Indications

**Figure 2.**
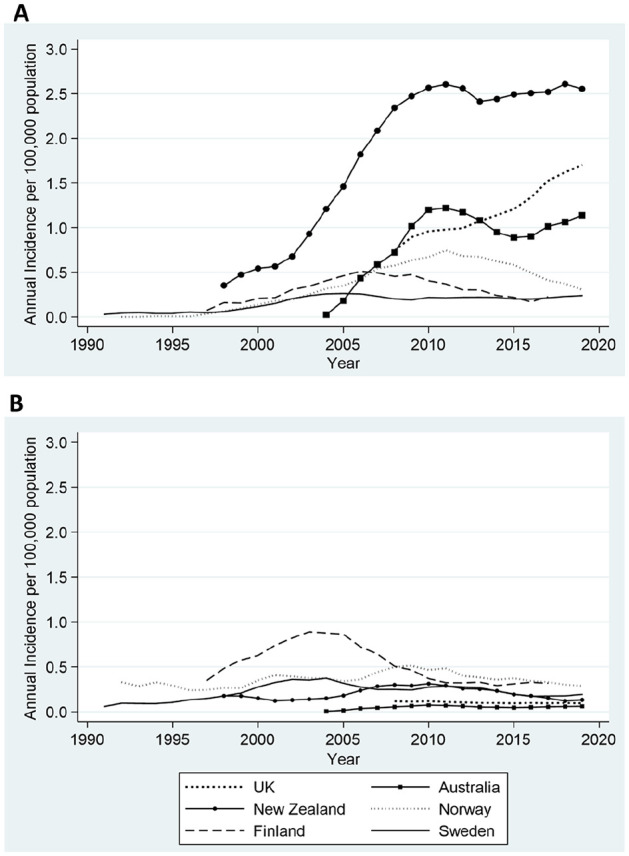
Annual incidence rates for primary ankle replacement by OA and RA disease indication categories for 6 countries. The data represent 5-year moving averages for (A) OA-specific and (B) RA-specific annual incidence rates of ankle replacement per 100 000 population for the entire registry period. OA, osteoarthritis; RA, rheumatoid arthritis.

We also examined the annual incidence of ankle replacements by indication categories, specifically, OA and RA (Figure 2). We wanted to examine whether differences in absolute (all diagnoses) incidence rates for ankle replacements were driven by changes in OA and RA disease indications. There were marked differences between countries in OA and RA-specific annual incidence with OA the dominant indication for surgery in the United Kingdom, Australia, and New Zealand. In contrast, in Finland, Sweden, and Norway the incidence of RA-diagnosed ankle replacements was greater than for the United Kingdom, Australia, and New Zealand. The relative proportions of OA and RA varied between countries as did the trend in these proportions over time. New Zealand, Australia, Norway, and Finland demonstrated a rapid increase in the annual incidence of OA-diagnosed ankle replacements in approximately the first 5-10 years of registry inception, though beyond the year of peak incidence, temporal trends were highly divergent. These initial rises in ankle replacement incidence were thought to be related to increasing data completeness and the speed of registry adoption. The United Kingdom showed a continuous increase in incidence of OA whereas growth in Sweden has remained stable over time (Figure 2). In contrast, there were marked differences between all countries for the annual incidence of RA-diagnosed ankle replacements. The annual incidence of RA-specific ankle replacements remained low and steady in both the United Kingdom and Australia over time. In contrast, RA rates in Finland, Norway, Sweden, and New Zealand declined after year of peak incidence.

Taking into consideration the entire registry period, New Zealand had the greatest annual incidence of OA-diagnosed ankle replacements per 100 000 population whereas Sweden had the lowest annual incidence (Table 4). Similarly, for the common registry period where all registries had data, New Zealand had the greatest annual incidence of OA-diagnosed ankle replacements per 100 000 population compared with Sweden, which had the lowest incidence.

**Table 4.** Annual incidence rates for primary ankle replacement by OA and RA indications.

Country	Annual Incidence Rates for Primary Ankle Replacement per 10^5^ population						
	Years Included	Disease Indication	5-Year Moving Averages for Entire Registry Period, Mean ± SD	5-Year Moving Averages for Overlapping Period^a^, Mean ± SD	5-Year Moving Averages After Year of Peak Incidence^b^		
					0-4 y	5-9 y	≥10 y
Australia	2006-2019	OA	0.9 ± 0.4	1.1 ± 0.1	1.1	1.03	–
		RA	0.1 ± 0.02	0.1 ± 0.01	0.1	0.1	–
Finland	1999-2015	OA	0.3 ± 0.1	0.3 ± 0.1	0.5	0.3	0.2
		RA	0.5 ± 0.2	0.3 ± 0.03	0.6	0.3	0.3
New Zealand	2000-2019	OA	1.8 ± 0.9	2.5 ± 0.1	2.5	2.5	–
		RA	0.2 ± 0.1	0.3 ± 0.04	0.3	0.2	–
Norway	1994-2019	OA	0.3 ± 0.3	0.7 ± 0.1	0.7	0.5	0.3
		RA	0.4 ± 0.1	0.4 ± 0.1	0.5	0.3	0.3
Sweden	1993-2019	OA	0.2 ± 0.1	0.2 ± 0.01	0.3^c^	0.2^c^	0.2^c^
		RA	0.2 ± 0.1	0.3 ± 0.04	0.3^c^	0.3^c^	0.2^c^
United Kingdom	2010-2019	OA	1.2 ± 0.3	1.1 ± 0.1	1.0^d^	1.5^d^	–
		RA	0.1 ± 0.01	0.1 ± 0.01	0.1^d^	0.1^d^	–

Abbreviations: OA, osteoarthritis; RA, rheumatoid arthritis.

a Overlapping period is defined as the period of 2010-2015 where all 6 registries had data.

b Year of peak annual incidence was based on raw annual incidence rates per 10^5^ population for all diagnoses.

c Sweden had 3 peaks, and so we used the first peak (2003) as the year of peak incidence.

d The United Kingdom did not show peak incidence, and so we present 5-year moving averages from registry inception.

## Discussion

In the present study, we examined between-country differences in ankle replacement utilization and observed marked between-country differences in annual incidence, rates of speed of adoption, changes in indications for surgery, and in demographics of patients undergoing ankle replacement. Through examining between-country differences in ankle replacement utilization, in the absence of outcome data, we can begin to describe how far current demands are being met.

Few studies have examined temporal trends in the utilization of TAR,^45,46,58^ with one study, using joint registry data, identifying 3 time trends^45^ and a further study reporting an increase in US ankle replacements between 1997-2003 and 2004-2010.^46^ Between-country differences in ankle replacement incidence may exist as a result of differences in data collection processes^6,43^ rather than varying rates of disease burden.

We observed an initial, greater than linear growth in use of ankle replacements in 5 of the 6 registries and after year of peak incidence, there was no consistent trend in utilization (eg, continuous decline, decline followed by a secondary growth, plateau, etc). The United Kingdom, which has shown continuous growth in ankle replacement utilization, was the only exception. This pattern of utilization in the early years of registry inception was expected, owing to previous comparative assessments of joint registries, and was thought to be a consequence of increasing data completeness and surgical familiarity.^10,14,43^ Other factors that are likely to affect estimation of ankle replacement incidence are the temporal trends in speed of data collection or of uptake in surgery.

After each country’s data had been standardized to its respective population, New Zealand was found to have the greatest number of hospitals and surgeons performing ankle surgery per year whereas Sweden had the lowest. Although it is highly likely that number of hospitals and surgeons available to perform ankle replacements would likely influence incidence, these factors did not appear to correlate with ankle replacement incidence. Finland had a greater number of hospitals performing ankle surgery compared to Norway yet annual ankle incidence was larger in Norway. More so, it is likely that surgical demand drives the need for increased number of hospitals/surgeons performing ankle replacement.

Changes in disease indications are also likely to influence temporal trends in absolute ankle replacement incidence. With the introduction of biological therapy, there has been a decline in rates of RA-related joint replacements.^15,16^ Annual incidence rates for RA-specific ankle replacements remained relatively steady in the United Kingdom and Australia, and similarly, rates remained stable in New Zealand though it declined from the year of peak incidence. In contrast, annual rates of RA-specific ankle replacements declined marginally in Norway and Sweden, though more substantially in Finland. One strong possibility is that improved RA management, combined with a decline in the prevalence of RA,^1^ has caused a decline in the need of surgical treatment of RA.^7,15,23^ It is acknowledged that RA can lead to surgery because of secondary OA: the coding of which varies between countries. Our assumption that such patients with underlying RA as the reason for their joint replacement would be categorized as having an RA-diagnosed ankle replacement was not testable in these data. These data suggest that changes in disease indications do not translate to changes in absolute (all diagnoses) ankle replacement incidence rates.

The annual incidence of ankle replacement will vary by population size and demographic structure. We have focused on the rates in adults (aged ≥18 years) to give an indication of the relative quantum of surgery in these countries. There is variation in the age structure of these populations with New Zealand, for example, having the lowest and Finland the highest proportion aged ≥65 years.^40^ Thus, even without formal age adjustment, the between-country differences do not appear to be explained by age.

There are several strengths to this study. We were able to utilize data from all the national joint registries with ankle data for a 25-year period extending to 2019. Even among the 6 registries studied, the long period of observations allowed us to identify different time trends in the annual incidence of ankle replacements. We examined a host of registry-level and demographic factors which were likely to influence ankle replacement incidence. Further, in order to minimize the effects of surgical learning and data completeness on our estimates of ankle replacement incidence, we also examined changes in annual incidence rates for all diagnosis, OA-specific, and RA-specific procedures from the peak on annual incidence.

There are several potential limitations to this study. First, we obtained aggregate data, rather than patient-level data, from the joint registries. In general, national registries do not provide primary data for researcher analysis. It is not possible to make any inference about the quality of the aggregated data provided. and we have assumed that the appropriate error, range, and consistency checks were made at a local level and that such issues have not affected the conclusion. Second, although we attempted to harmonize primary indications for ankle replacement, it is very challenging to compare the relative indications for surgery given the absence of an agreed system for classifying patients. In part, this is due to the arbitrary nature of how the pathway to end-stage disease is considered within these registries. Thus, individuals with OA secondary to RA may be coded as either disease. More relevant perhaps to these data is the lack of an agreed approach to the role of trauma. There is a strong evidence that ankle OA follows on from major trauma,^27^ but after a varying interval of time. Whether trauma or OA are used as the primary terms on each register, or even within a register by different contributing units, is unknown. This is important in understanding the underlying explanation for the observed differences in the proportions with these disorders. One conclusion from our data review and a future work, therefore, is that it would be useful for there to be an international agreement, perhaps sponsored by bodies such as the International Society of Arthroplasty Registries, to harmonize causal coding for this joint replacement.

Third, we did not evaluate patient-reported outcome measures in this study. Most national joint registries have detailed data from knee and hip joint replacement with greater emphasis on outcomes and complications. Given their lower frequency, there is much less attention given to these aspects on outcomes of other joint sites including the shoulder, elbow, and ankle. Indeed, there is a much greater number of hip and knee registries worldwide.^28^ The wide variation in incidence, trends and indications for ankle replacement in these registries highlights the need for more nationally collected and reported data on outcomes. The robust interpretation of any differences in adverse outcomes and implant survival will need to take account of many of the aspects already referred to, such as use of diagnostic labels, differences in indication, and the changing epidemiology over time. Future work should include harmonization of such outcome measures^44^ to allow between-registry comparisons examining safety of joint replacements. Lastly, and importantly, reporting of ankle replacements was not mandatory in all registries. The estimates provided herein should be seen as lower limits, as ankle replacements could have been done that were not recorded in some of the registries.

### Conclusions

In these 6 countries with national registries for ankle replacement, we found marked differences with the utilization of ankle replacements. Such differences are likely driven not only by variation in annual incidence rates and differences in the main indications for surgery but also due to differences in the registries’ inception dates and data capture processes. Standardizing national registry procedures would enable ankle replacement data to be collected consistently and support future international comparisons.

## Supplementary Material

### Registries

#### National Joint Registry for England, Wales, Northern Ireland, the Isle of Man and the States of Guernsey (UK-NJR)

The UK-NJR, the largest orthopedic registry in the world with >3 million records,^32^ was created in 2002 and aimed to capture data on all hip and knee replacement operations in England and Wales; national coverage was achieved when Northern Ireland later jointed in 2013 and the Isle of Man in 2015.^34^ Data collection is mandatory for all NHS trusts and foundations in the United Kingdom (England, Wales, and Northern Ireland) and for all independent sector hospitals in England and Wales. Capture of ankle replacements first started in April 2010. Data on ankle replacements are available from published annual reports^32^ or through a publicly available online data management portal^35^; here, we extracted data from the online portal as it was more detailed than the annual reports though data were only available from April 1, 2010, to December 31, 2019, whereas the 2020 annual report includes more recent data up until February 29. 2020.^32^ Data completeness is assessed through examination of 3 indicators of data quality, which include compliance, consent, and likability.^31^ Completeness for knee, hip, ankle, elbow, and shoulder procedures are compared to reports submitted to the Hospital Episodes Statistics (HES) service in England and to the Patient Episode Database Wales (PEDW) service in Wales; this does not include independent sector hospitals. In the current study, we used percentage consented at time of operation (cumulative for all joints)^33^ as our measure of data completeness. For some years, completeness was reported as 0% due to a single NHS hospital reporting 0% consented at time of surgery.

#### Australian Orthopaedic Association National Joint Replacement Registry (AOANJRR)

The AOANJRR started knee and hip arthroplasty data collection in South Australia in 1999; national coverage, including all states and territories, was achieved in 2002.^2^ In 2006, the registry expanded its data collection to include the ankle joint with full, national coverage achieved in late 2007 (November). Data from both public and private hospitals is validated through comparison against data provided to the state and territory health departments. Hospital participation was nonobligatory; nevertheless, all hospitals undertaking joint replacements have reportedly agreed to participant in data collection. Our study included all primary total ankle replacement procedures reported to the AOANJRR between January 1, 2008, and December 31, 2019.

#### Finland Arthroplasty Register (FAR)

We used data from the Finnish Arthroplasty Register (FAR). As part of registry procedures, FAR has captured nearly all hip and knee replacement procedures performed since 1980 with 45 departments contributing data.^38^ Registration of all joint replacements, which was voluntary at registry inception, became obligatory in 1997 and now all orthopedic centers registered with FAR are obliged to provide patient data to the Finnish National Institute for Health and Welfare.^41^ All ankle replacement data are currently gathered using paper forms and has not been translated to an online platform^12^; therefore, we requested all relevant ankle data directly from the registry. Crude estimation of data completeness first started in 1996, ankle registry inception started in 1980, with all ankle replacements reported to FAR compared against the Finnish Hospital Discharge Register (HDR).

#### The Norway Arthroplasty Register (NAR)

The National Arthroplasty Register of Norway was first established in 1987 with a primary focus on capturing data on hip replacements. In 1994, registration was extended to include replacement procedures of all joints including the ankle. Whereas it is not compulsory to report joint replacements, compliance has been reported as high as 95% for recent years.^59^ Data completeness has been reported previously for the period 1999 to 2002.^10^ In brief, completeness was assessed by comparison of the number of ankle replacements reported to the NAR, which is voluntary, against counts reported to the Norwegian Patient Register (NPR); reporting to the NRP is mandatory, with electronic administrative patient records from all hospitals sent to the NRP. In the currents study, data on annual completeness was provided for the period of 2008 to 2018: data reported are for the completeness of primary ankle replacements only.

#### New Zealand Orthopaedic Association (NZOA) Arthroplasty Register

New Zealand’s national arthroplasty register was established in 1997 capturing data on all knee and hip replacement procedures.^36^ In January 2000, the registry was expanded to include the collection of all total joint replacements for ankles within the whole country. The registry routinely achieves a compliance rate of at least 90% for all hospitals undertaking joint replacement surgery in New Zealand.^60^ Data compliance is assessed annually for public hospitals through comparing all joint replacement surgeries to the NZ Health Information Service (NZHIS).

#### The Swedish Ankle Registry (SwedAnkle)

Since 1997, the Swedish Ankle Registry has captured data on all national ankle replacements (within 17 units), and from 2008, the registry expanded collection to include data on all ankle fusions and supramalleolar osteotomies.^57^ The Swedish registry consists of 3 completely separate ankle registries: (1) primary replacements and revisions/reoperations, (2) primary fusions and re-arthrodesis, and (3) supramalleolar osteotomies. Whereas the Swedish ankle registry was introduced 1997, cases of ankle replacements from 1993 to 1996 were registered retrospectively. Submission of ankle data by surgeons and patients is voluntary. The aforementioned registries have published all annual reports in English with the exception of Sweden for the years 2009 to 2011.^57^

## Supplemental Material

sj-docx-1-fai-10.1177_10711007211012947 – Supplemental material for Trends in the Utilization of Ankle Replacements: Data From Worldwide National Joint Registries Supplemental material, sj-docx-1-fai-10.1177_10711007211012947 for Trends in the Utilization of Ankle Replacements: Data From Worldwide National Joint Registries by Thomas A. Perry, Alan Silman, David Culliford, Lucy Gates, Nigel Arden and Catherine Bowen in Foot & Ankle International

sj-docx-2-fai-10.1177_10711007211012947 – Supplemental material for Trends in the Utilization of Ankle Replacements: Data From Worldwide National Joint Registries Supplemental material, sj-docx-2-fai-10.1177_10711007211012947 for Trends in the Utilization of Ankle Replacements: Data From Worldwide National Joint Registries by Thomas A. Perry, Alan Silman, David Culliford, Lucy Gates, Nigel Arden and Catherine Bowen in Foot & Ankle International

sj-pdf-1-fai-10.1177_10711007211012947 – Supplemental material for Trends in the Utilization of Ankle Replacements: Data From Worldwide National Joint Registries Supplemental material, sj-pdf-1-fai-10.1177_10711007211012947 for Trends in the Utilization of Ankle Replacements: Data From Worldwide National Joint Registries by Thomas A. Perry, Alan Silman, David Culliford, Lucy Gates, Nigel Arden and Catherine Bowen in Foot & Ankle International
