# Supplementary material for: Trends in the Utilization of Ankle Replacements: Data From Worldwide National Joint Registries
Source: Foot Ankle Int. 2021 Jun 17;42(10):1319–29. doi: 10.1177/10711007211012947 (PMC8521348; doi:10.1177/10711007211012947)
Supplement: sj-docx-1-fai-10.1177_10711007211012947 – Supplemental material for Trends in the Utilization of Ankle Replacements: Data From Worldwide National Joint Registries [file sj-docx-1-fai-10.1177_10711007211012947.docx]

| **Supplementary Table 1:** Primary ankle procedures captured across the included national joint registries. | |
| --- | --- |
| **Country/Registry** | **Primary Ankle Replacements Included** |
| UK | Primary total prosthetic replacement using or not using cement  Primary total prosthetic replacement not classified elsewhere (e.g. hybrid) |
| Australia | Total ankle replacement: defined as replacement of all (total) of the articular surface |
| New Zealand | Total ankle replacements |
| Finland | Total ankle replacements |
| Sweden | Registry only contains uncemented cases |
| Norway | Primary partial or total prosthetic replacement of ankle joint using or not using cement  Primary total prosthetic replacement of ankle joint using hybrid technique  Primary total prosthetic replacement of ankle joint using cement |
