# Supplementary material for: Trends in the Utilization of Ankle Replacements: Data From Worldwide National Joint Registries
Source: Foot Ankle Int. 2021 Jun 17;42(10):1319–29. doi: 10.1177/10711007211012947 (PMC8521348; doi:10.1177/10711007211012947)
Supplement: sj-docx-2-fai-10.1177_10711007211012947 – Supplemental material for Trends in the Utilization of Ankle Replacements: Data From Worldwide National Joint Registries [file sj-docx-2-fai-10.1177_10711007211012947.docx]

| **Supplementary Table 2:** Classification of disease indications for primary ankle replacements. | | | | |
| --- | --- | --- | --- | --- |
|  | **Primary Disease Indication** | | | |
| **Country** | **Osteoarthritis** | **Rheumatoid arthritis** | **Trauma** | **Other** |
| UK | OA | RA | - | Other inflammatory arthropathy, ‘Other’ |
| Australia | OA | RA | Fracture/Dislocation | Other Inflammatory Arthritis, Instability, Osteonecrosis, Tumour, ‘Other’ |
| New Zealand | OA | RA | Post old trauma | ‘Other’, Avascular necrosis |
| Finland | Primary arthroses | RA | - | ‘Other’ |
| Sweden | Primary OA | RA | Post-traumatic OA | Psoriatic arthritis, Hemochromatosis, ‘Other’ |
| Norway | Osteoarthritis | RA | Sequelae after fracture  Sequelae ligament tear  Acute fracture | Ankylosing spondylitis, Sequelae of infection, ‘Other’, Missing |
| Abbreviations: osteoarthritis (OA), rheumatoid arthritis (RA). | | | | |
